# Analysis of cholesterol-recognition motifs of the plasma membrane Ca^2+^-ATPase

**DOI:** 10.1007/s10863-024-10010-5

**Published:** 2024-03-04

**Authors:** Blanca Delgado-Coello, Ismael Luna-Reyes, Kevin M. Méndez-Acevedo, Jorge Bravo-Martínez, Danai Montalvan-Sorrosa, Jaime Mas-Oliva

**Affiliations:** 1 Departamento de Bioquímica y Biología Estructural, Instituto de Fisiología Celular, Universidad Nacional Autónoma de México, Apdo. Postal 70-243, Ciudad de México, C.P. 04510 México; 2 Departamento de Fisiología, Facultad de Medicina, Universidad Nacional Autónoma de México, Ciudad de México, México; 3 Departamento de Química de Biomacromoléculas, Instituto de Química, Universidad Nacional Autónoma de México, Ciudad de México, México; 4 Present Address: University of Cambridge Metabolic Research Laboratories and NIHR Cambridge Biomedical Research Centre, Wellcome-MRC Institute of Metabolic Science, Addenbrooke’s Hospital, Cambridge, UK

**Keywords:** CRAC/CARC motifs, Cholesterol, Plasma membrane Ca^2+^-ATPase, PMCA regulation, Molecular dynamics simulations, Lipid rafts

## Abstract

The plasma membrane Ca^2+^-ATPase (PMCA) is crucial for the fine tuning of intracellular calcium levels in eukaryotic cells. In this study, we show the presence of CARC sequences in all human and rat PMCA isoforms and we performed further analysis by molecular dynamics simulations. This analysis focuses on PMCA1, containing three CARC motifs, and PMCA4, with four CARC domains. In PMCA1, two CARC motifs reside within transmembrane domains, while the third is situated at the intracellular interface. The simulations depict more stable RMSD values and lower RMSF fluctuations in the presence of cholesterol, emphasizing its potential stabilizing effect. In PMCA4, a distinct dynamic was found. Notably, the total energy differences between simulations with cholesterol and phospholipids are pronounced in PMCA4 compared to PMCA1. RMSD values for PMCA4 indicate a more energetically favorable conformation in the presence of cholesterol, suggesting a robust interaction between CARCs and this lipid in the membranes. Furthermore, RMSF analysis for CARCs in both PMCA isoforms exhibit lower values in the presence of cholesterol compared to POPC alone. The analysis of H-bond occupancy and total energy values strongly suggests the potential interaction of CARCs with cholesterol. Given the crucial role of PMCAs in physiological calcium regulation and their involvement in diverse pathological processes, this study underscores the significance of CARC motifs and their interaction with cholesterol in elucidating PMCA function. These insights into the energetic preferences associated with CARC-cholesterol interactions offer valuable implications for understanding PMCA function in maintaining calcium homeostasis and addressing potential associated pathologies.

**Supplementary Information:**

The online version contains supplementary material available at 10.1007/s10863-024-10010-5.

## Introduction

Eukaryotic cells possess specialized proteins to maintain intracellular calcium concentration at nanomolar levels, with the plasma membrane Ca^2+^-ATPase (PMCA) playing a crucial role in calcium extrusion and fine-tuning. The topological model of PMCA originally based on that proposed for the sarco/endoplasmic calcium ATPase (SERCA), and confirmed later using cryo-EM of PMCA1 (Toyoshima et al. 2000; Gong et al. 2018), depicts a protein containing ten transmembrane domains (TM) connected by short extracellular loops. On the intracellular side, two loops harbor important sites; the first loop contains a site recognizing phospholipids and one of two sites recognizing the autoinhibitory calmodulin-binding domain (CBD), which, in resting conditions, maintains the ATPase in an autoinhibited state. The second loop has another site recognizing CBD and the catalytic site where the enzyme is phosphorylated at high intracellular Ca^2+^ concentrations. The C-terminus harbors the regulatory region where the calmodulin-binding site is located.

The four basic PMCA isoforms (PMCA1-PMCA4) are encoded by four different genes (*ATP2B1-ATPB2B4*) that by alternative splicing at their sites A and C produces over 20 splice variants (Shull and Greeb 1988; Strehler et al. 1989). As the site C encodes the calmodulin-binding site, variants edited at this site show different affinities for calmodulin and calcium being distributed differentially according to the tissue type (Caride et al. 1999). PMCA1 and PMCA4 are ubiquitously expressed, whereas PMCA 2 and PMCA3 are more abundant in the excitable tissues (Stauffer et al. 1993, 1995). The PMCA isoforms expressed in non-excitable and excitable tissues display low (slow isoforms) and high calcium affinities (fast isoforms), respectively (Caride et al. 2001; Zylinska and Soszynski 2000; Strehler et al. 2007).

The complexity of PMCAs regulation is further increased by various mechanisms influencing their activity. Most PMCAs are regulated by calmodulin, except in the liver, where hormones inhibit it by mechanisms mediated by heterotrimeric G proteins (Kessler et al. 1990; Lotersztajn et al. 1992; Delgado-Coello et al. 2006). Alternatively, calcium ATPases can be modulated by acidic phospholipids and cholesterol (Ansah et al. 1984; Brodin et al. 1992; Lopreiato et al. 2014).

As a whole, cholesterol influences in an indirect fashion, the fluidity, thickness, and permeability of the plasma membrane or it can directly regulate the activity and location of membrane proteins (Yeagle 1991; Jafurulla and Chattopadhyay 2017; Conrard and Tyteca 2019; Rivel et al. 2019; Garcia et al. 2019; Zakany et al. 2020). Our research group has previously studied the thermal stabilizing effect of cholesterol on the PMCA located in erythrocytes and cardiac muscle (Santiago-García et al. 2000; Mas-Oliva and Delgado-Coello 2007; Mas-Oliva and Santiago-García 1990; Ortega et al. 1996). These phenomena might be further associated with cholesterol-rich domains, such as lipid rafts, where PMCA has been identified in several tissues (Sepúlveda et al. 2006; Jiang et al. 2007, 2012; Fujimoto 1993; Xiong et al. 2009; Delgado-Coello et al. 2017). However, the mechanism governing the interaction between cholesterol and the different PMCA isoforms remains unclear.

In this study, we aimed to identify cholesterol recognition/interaction amino acid consensus (CRAC) sequences or the inverse sequence (CARC) in human and rat PMCAs, and to determine whether there is a direct interaction between cholesterol and these motifs. CRAC/CARC sequences are two of at least three possible domains present in membrane proteins capable of interacting with cholesterol (Fantini et al. 2016). So far, only one report has referred to the possible presence of these motifs in the PMCA, however, it was attributed to palmitoylation signals which determine the association between cholesterol and the PMCA4 isoform, due it was the only isoform identified in lipid raft domains (Epand 2006; Sepúlveda et al. 2006).

*In silico* techniques based on computational methods have emerged as valuable tools for investigating into membrane structure and its potential protein-protein interactions. This approach offers insights regarding specific residue contributions to cellular function in healthy states, as well as changes resulting from mutants and polymorphisms, and the identification of sites involved with therapeutic capability (Rajasekaran et al. 2008; Bhardwaj and Purohit 2020a, b). In the context of our research, the *in silico* approach is tailored towards probing protein-lipid interactions therefore, facilitating not only the exploration of various cholesterol-recognition motifs but also, the unveiling of the presence, exclusive to vertebrates, of CARC motifs within the tyrosine kinase receptor family (Cannarozzo et al. 2021).

CRAC motifs were first described in a peripheral-type benzodiazepine receptor containing the typical sequence: L/V-X_(1−5)_-Y-X_(1−5)_-R/K. Later they were also detected in proteins, such as caveolin and apolipoprotein A-I expressed in mice (Li et al. 1998). CRAC sites show a wide distribution in G-coupled receptors (Hanson et al. 2008; Jafurulla et al. 2011), in ABCG1/ABCG2 transporters (Sharpe et al. 2015; Gál et al. 2015), and in ion channels involved in store-operated Ca^2+^ entry mechanisms (Picazo-Juárez et al. 2011; Pacheco et al. 2016). They have also been identified in viral coat proteins, where a role in the membrane fusion with target host cells has been suggested (Luz-Madrigal et al. 2013). CRAC sequences are located in or close to transmembrane domains, or in the interface of the plasma membrane. Li et al. showed that L/V residues interact with the hydrophobic side chain of cholesterol, Y interacts with the 3’-OH group of cholesterol, while K/R amino acids contribute to form a binding site (Li et al. 1998). Moreover, there is evidence suggesting that CRAC motifs inside TM comply with features to properly fit the cholesterol molecule (Barrantes 2013). The interaction between cholesterol and proteins containing CRAC motifs has been shown using a minimal synthetic CRAC peptide -LWYIK- containing a cysteine in N- or C-ends to achieve the coupling to different lipids through disulfide bonds, showed that cholesterol is the preferred molecule in the neighborhood of liquid-disordered bilayers (Mukai et al. 2016).

Further analysis of the nicotinic acetylcholine receptor, led to the discovery of the inverted sequence or CARC motif: K/R-X_(1−5)_-Y/F-_(1−5)_-X-L/V, where the central amino acid can be Y or F (Baier et al. 2011; Di Scala et al. 2017). They are located in TM domains that interact with lipids. Upon comparison with other proteins, it appears that CARC sequences are indeed involved in interactions with cholesterol and it is suggested that the CRAC/CARC and tilted domains present in various membrane proteins share common molecular mechanisms for binding cholesterol (Baier et al. 2011).

In type I membrane proteins, whose N-terminus is located on the extracellular face, it is possible the coexistence in the same TM domain, of CARC motifs in the outer leaflet and CRAC motifs in the inner leaflet. In type 2 proteins, where the C-end is on the extracellular side, CARC and CRAC arrange in the opposite sense. These coexistent sequences participate in a type of interaction named “mirror code”, which would involve two symmetric cholesterol molecules (Fantini et al. 2016).

Although CRAC/CARC sequences show an affinity to stay at the membrane-water interface, it seems that the recognition of cholesterol occurs at the surface, but partial penetration into the bilayer is also required. In fact, the cholesterol molecule has a thermodynamic constraint that is related to the best orientation within a lipid bilayer, fitting well with its polar side oriented to the polar/apolar interface (Di Scala and Fantini 2017). Moreover, it has been demonstrated that CRAC motifs located in domains outside the lipid bilayer are not able to bind cholesterol or other sterols (Méndez-Acevedo et al. 2017).

In this study, we demonstrate only the presence of CARC motifs in human and rat PMCA isoforms. Based on our previous findings about the thermostability provided by cholesterol to PMCAs expressed in cardiac muscle and erythrocytes (Santiago-García et al. 2000; Mas-Oliva and Delgado-Coello 2007), and considering that PMCA1 the most abundant isoform in all tissues, except human erythrocytes where PMCA4 shows the highest expression (Stauffer et al. 1995; Strehler et al. 1990; Pászty et al. 1998; Zámbó et al. 2017), we conducted molecular dynamics simulations of both housekeeping isoforms. Thus, the discussion is focused on the possible relevance of the interaction between cholesterol and CARC sequences present in PMCA1 and PMCA4 isoforms.

## Materials and methods

### Analysis of CRAC/CARC sequences present in rat and human PMCA isoforms

The presence of CRAC and CARC motifs was determined using the ScanProsite database (Sigrist et al. 2013), using the UniProt IDs for human (hPMCA) and rat PMCA (rPMCA) isoforms, as indicated in Table 1. The alignment of the CRAC/CARC domains present in the four human PMCA isoforms and the comparison with rat PMCAs were performed using the UniProt (The Uniprot Consortium 2023) database tools (https://www.uniprot.org).

### Molecular dynamics simulations of human housekeeping PMCA isoforms

The model of human PMCA1 that not includes its C-end, was retrieved from the cryo-EM structure associated with neuroplastin, a novel obligatory subunit described for this isoform (PDB ID 6A69) (Gong et al. 2018). Human PMCA4 was predicted and modeled without restrictions and the template-based protein structure analysis was performed with the I-Tasser tools (UniProt IDs: P23634) (Yang and Zhang 2015). For further simulations, the model with the highest quality structure indicators was used.

Lipid bilayers were generated using the CHARM-GUI server (Jo et al. 2008) and a bilayer builder module. Bilayers were built using phosphatidylcholine (POPC) only for cholesterol-free systems, and POPC and 30% cholesterol of membrane area were included in cholesterol-containing systems, allowing an even distribution of cholesterol on both leaflets of the plasma membrane. Atomistic models were employed throughout all simulations, with the final system size ranging between 480,000 and 500,000 atoms. CHARMM36 force field (Huang and Mackerel 2013) and TIP3P water (Jorgensen et al. 1983) were utilized for molecular dynamics (MD) simulations. All systems were simulated in cubic boxes under periodic boundary conditions and incorporating long-range electrostatic interactions via the particle mesh Ewald summation method. Prior to equilibrium, a two-step minimization was conducted; the steepest descent method followed by the conjugate gradient method. To balance the solvent molecules and relax the structures, we equilibrated all systems using NVT ensemble followed by an NPT ensemble, at 303.15 °K and 1 atm. The Bussi-Donadio-Parrinello thermostat and the C-rescale barostat were employed for system equilibration. MD simulations were performed in a semi-discontinuous fashion using 1.5 ns restart points with 2 fs time steps, data production was carried out using Nose-Hoover thermostat and Parrinello-Rahman barostat; the resulting trajectories were merged for further analysis. Final trajectories had a total duration of 150 ns, and at least three replicas were performed for each system (Abraham et al. 2023). By means of GROMACS built-in tools the MD simulations analysis included: total energy determinations, partial lipid density profiles, the root mean square deviation (RMSD), and the root mean square fluctuation (RMSF) of the systems. All images of the PMCA simulations shown in this work were rendered using the Visual Molecular Dynamics (VMD) software (Humphrey et al. 1996).

## Results

### PMCA isoforms contain CARC motifs

**Fig. 1:**
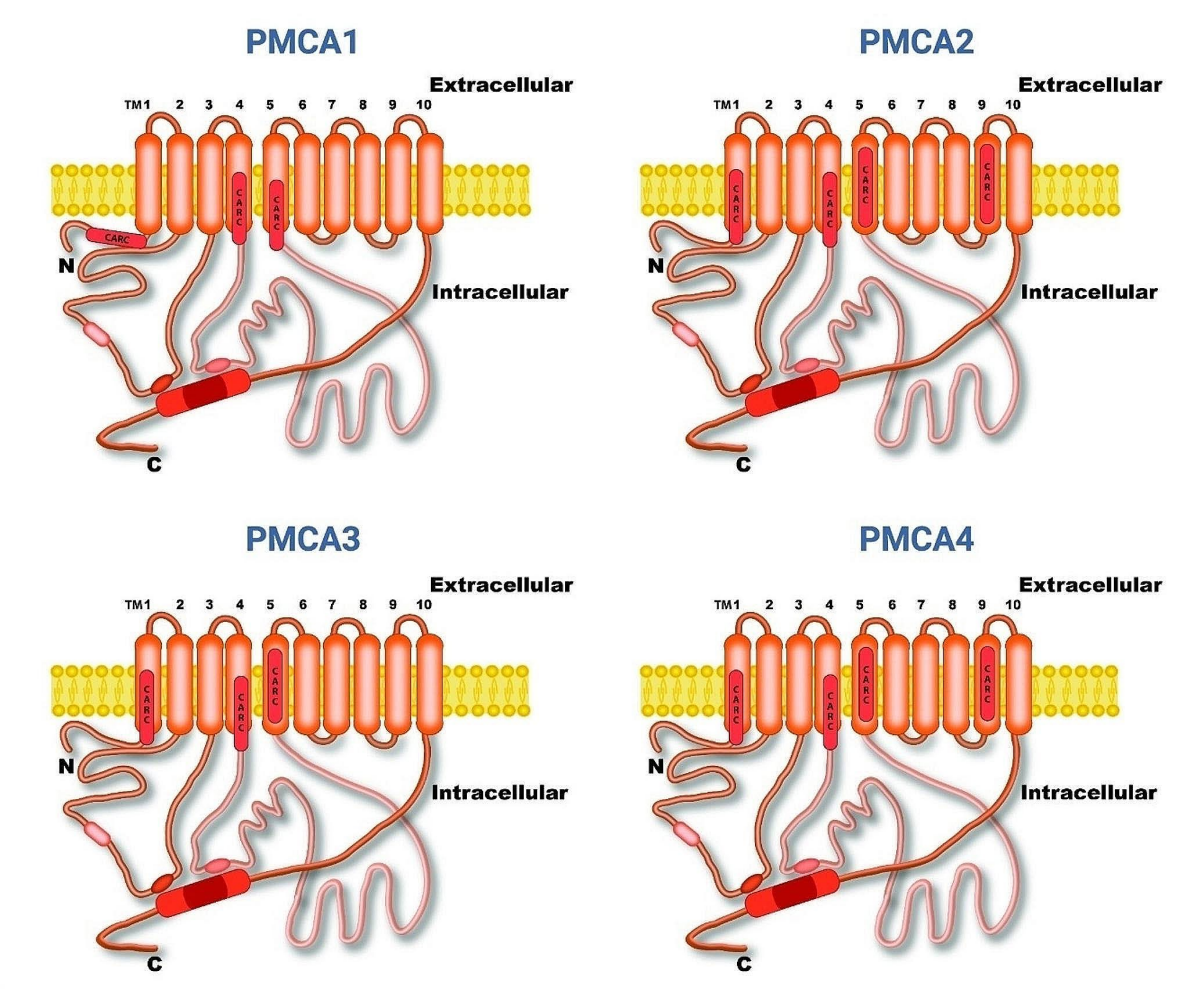
Topological location of CARC motifs in human/rat PMCA isoforms. The transmembrane domains indicating the predicted CARC sequences are highlighted in red. N: N-terminus; C: C-terminus; TM: Transmembrane domain

PMCAs comprise proteins with an approximate mass of 135 kD containing ten TM domains, and N- and C-ends located on the cytoplasmic side of the cell membrane (Table 1). Our analysis of consensus sequences for cholesterol recognition motifs shows that in all human/rat PMCA isoforms, only CARC sequences, that we named CARC1-CARC4, are present. The CARC1 motif of PMCA1 is close to the TM1; while the other CARCs present in both PMCA isoforms are partially or completely embedded in the inner membrane leaflet, to note is that CARC3 and CARC4 motifs of PMCA4, are completely embedded in their respective TM domains (Fig. 1).

**Table 1:** Amino acids comprised in TM domains of human and rat PMCA isoforms

	TM1	Isoform	TM3	TM4	TM5	TM6	TM7	TM8	TM9	TM10
hPMCA1	106–126	155–175	367–386	419–439	856–876	883–903	928–948	972–991	1006–1027	1040–1060
hPMCA2	95–115	153–173	391–410	444–461	876–895	906–926	947–969	988–1009	1029–1050	1061–1082
hPMCA3	98–118	156–176	365–384	418–435	850–869	880–900	921–943	962–983	1003–1024	1035–1056
hPMCA4	93–113	151–171	357–376	410–427	841–860	871–891	912–934	953–974	994–1015	1026–1047
rPMCA1	106–126	155–175	367–386	419–439	856–876	883–903	928–948	972–991	1006–1027	1040–1060
rPMCA2	95–115	153–173	391–410	444–461	876–895	906–926	947–969	988–1009	1029–1050	1061–1082
rPMCA3	98–118	156–176	365–384	418–435	850–869	880–900	921–943	962–983	1003–1024	1035–1056
rPMCA4	93–113	151–171	357–376	410–427	841–860	871–891	912–934	953–974	994–1015	1026–1047

UniProt IDs: hPMCA1 (P20020); hPMCA2 (Q01814); hPMCA3 (Q16720); hPMCA4 (P23634); rPMCA1 (P11505); rPMCA2 (P11506); rPMCA3 (Q64568); rPMCA4 (Q64542)

**Fig. 2:**
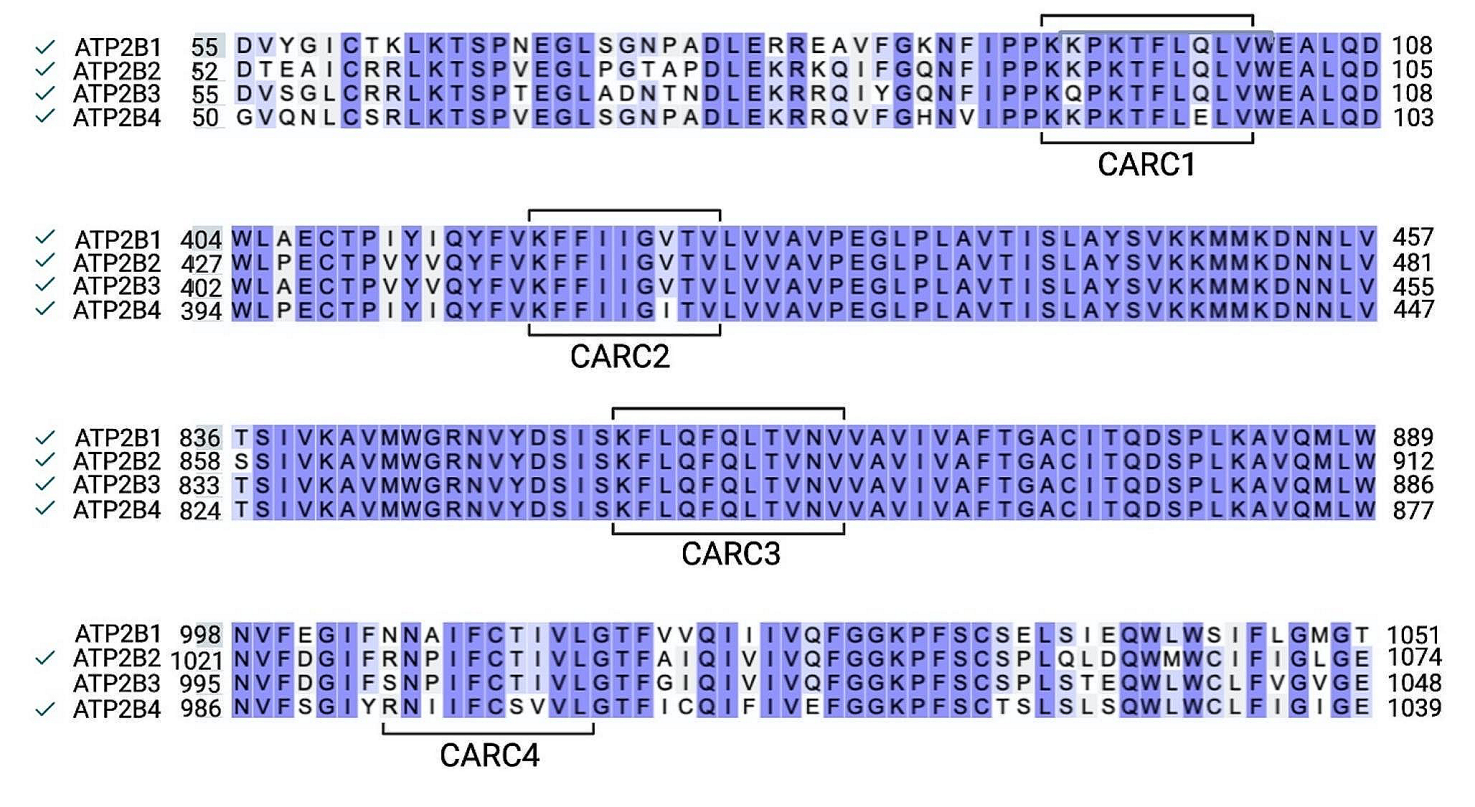
Alignment of CARC sequences present in human PMCA isoforms: PMCA1 (*ATP2B1*); PMCA2 (*ATP2B2*); PMCA3 (*ATP2B3*); PMCA4 (*ATP2B4*). The residues comprising CARC sequences are marked with brackets; on the left side, the isoforms that contain the corresponding CARC domains are indicated (✓). Note that CARC4 is only present in PMCA2 and PMCA4 isoforms. Purple boxes highlight conserved sequences among the different PMCA isoforms

According to the preliminary alignments performed (not shown), the homology between human and rat PMCAs is high, reaching 99% for PMCA1, approximately 95% for PMCA2 and PMCA3, and 87% for PMCA4. The lengths of the sequences of h/r-PMCA1 (1220 amino acids) and h/rPMCA2 (1243 amino acids) are equal, whereas PMCA3 (hPMCA3 1220 aa vs. 1258 aa for rPMCA3) and PMCA4 (hPMCA4 1241 aa vs. 1203 aa for rPMCA4) isoforms differ in about 38 amino acids on their C-end. However, the CARC sequences for both species show the same location along their respective sequences (Table 2). Furthermore, a perfect homology is observed among the corresponding CARCs of h/rat PMCA1 and PMCA4 isoforms (Table 3). Thus, for simplicity, from now on we only present data from human PMCAs; the alignment of CARC domains is shown in Fig. 2.

**Table 2:** Amino acids comprised in the CARC motifs of h/r PMCAs

Isoform	CARC1	CARC2	CARC3	CARC4
PMCA1	93–102	418–426	854–864	-
PMCA2	90–99	442–450	877–887	1028–1037
PMCA3	93–102	416–424	851–861	-
PMCA4	88–97	408–416	842–852	993–1002

**Table 3:** Comparison of CARC sequences of h/r PMCA1/PMCA4 isoforms

Isoform	CARC number	Sequence
hPMCA1	1 (93–102)^*^	Kkpkt.Flql.V
rPMCA1	1 (93–102)	Kkpkt.Flql.V
hPMCA1	2 (418–426)^*^	Kf….FiigvtV
rPMCA1	2 (418–426)	Kf….FiigvtV
hPMCA1	3 (854–864)^*^	Kflq.FqltvnV
rPMCA1	3 (854–864)	Kflq.FqltvnV
hPMCA4	1 (88–97)^*^	Kkpkt.Flel.V
rPMCA4	1 (88–97)	Kkpkt.Flel.V
hPMCA4	2 (408–416)^*^	Kf….FiigvtV
rPMCA4	2 (408–416)	Kf….FiigvtV
hPMCA4	3 (842–852)^*^	Kflq.FqltvnV
rPMCA4	3 (842–852)	Kflq.FqltvnV
hPMCA4	4 (993–1002)^*^	Rnii.Fcsvv.L
rPMCA4	4 (993–1002)	Rnii.Fcsvv.L

^*^ CARC sequences analyzed in molecular dynamics simulations

### Molecular dynamics simulations of human PMCA1 and PMCA4 isoforms

**Fig. 3:**
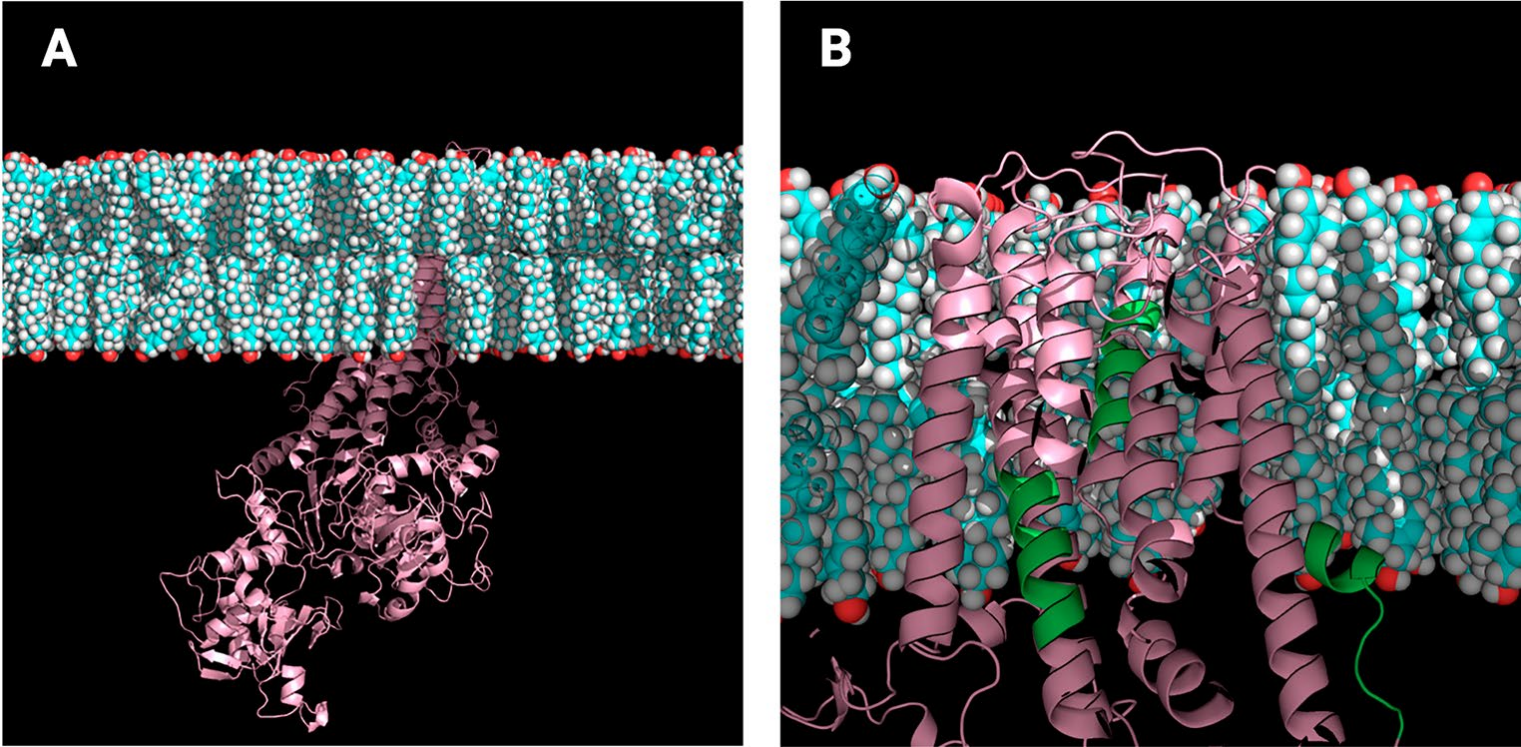
Molecular dynamics simulation of PMCA1. (**A**) Landscape view of a simulated system where PMCA 1 (pink) is shown in a membrane containing cholesterol (cyan) and POPC; water molecules, ions, and POPC, were removed from the image. (**B**) Close-up image of the transmembrane domains of PMCA1, where CARC motifs are indicated in green

**Fig. 4:**
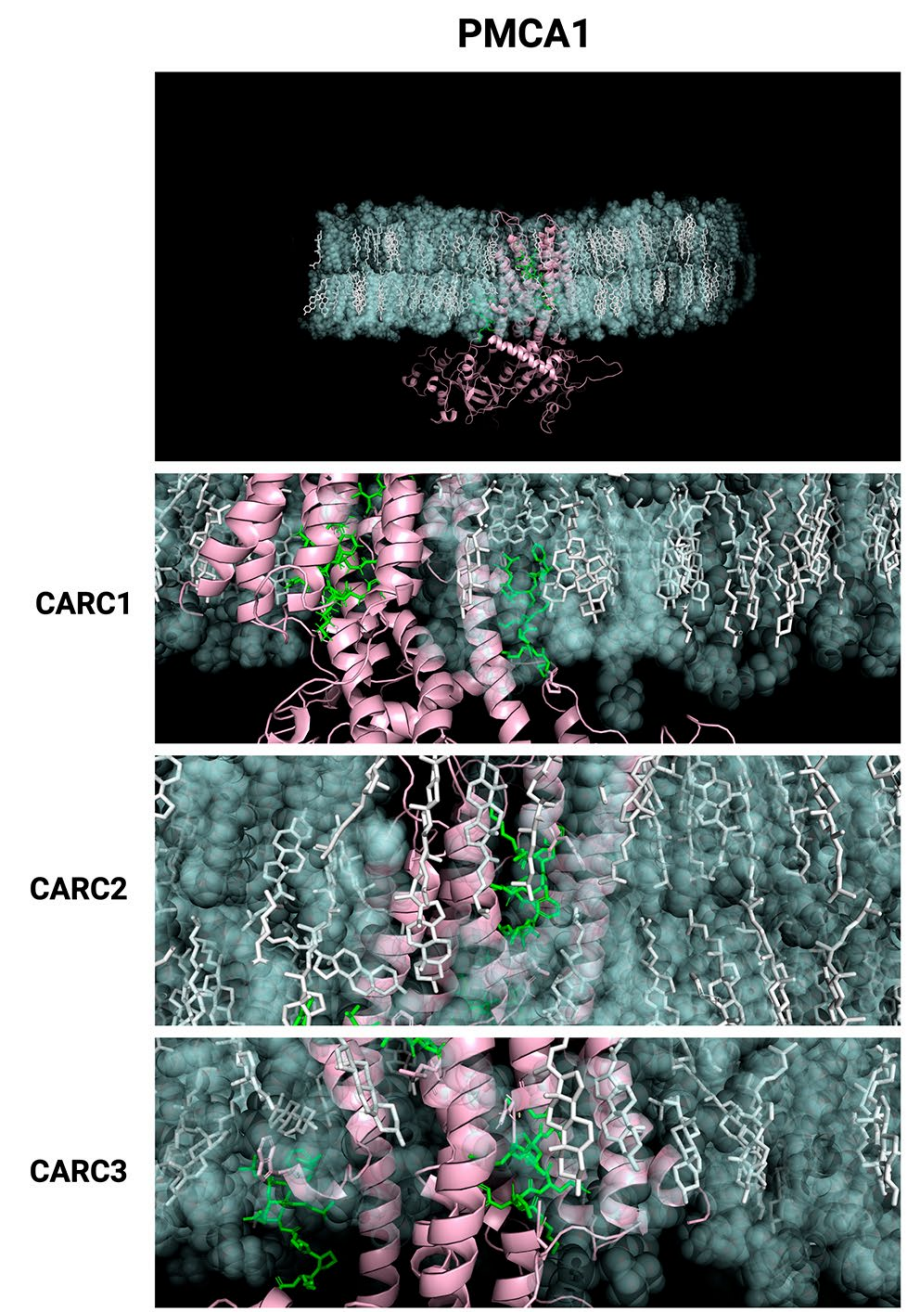
Close-up images of CARC motifs found in PMCA1. In the top figure, the landscape view of CARC motifs present is shown. The close-up images of CARC1-CARC3 motifs are shown as green licorice lines, cholesterol molecules are presented as white licorice lines, surrounding POPC molecules are presented as transparent green beads, and non-CARC-associated protein depicted as pink ribbons

Once we identified only CARC motifs in the PMCAs, we performed MD simulations of the complete proteins and a further analysis to determine possible interactions of these sequences with the surrounding cholesterol molecules. The human PMCA1 isoform has three CARC motifs; the corresponding MD simulation is shown in Fig. 3. In a close-up view, it is possible to observe two CARC motifs partially included in the transmembrane domains and one more in the interface towards the intracellular side (Fig. 3B). For a better understanding of CARCs motifs in the context of the simulations, we rendered a general view of PMCA1 embedded in the membrane with specific focus on each CARC motif (Fig. 4). These images show the location of CARC motifs in or close to TM in the inner leaflet where charged residues interact with the hydroxyl group of cholesterol and other lipids.

**Fig. 5:**
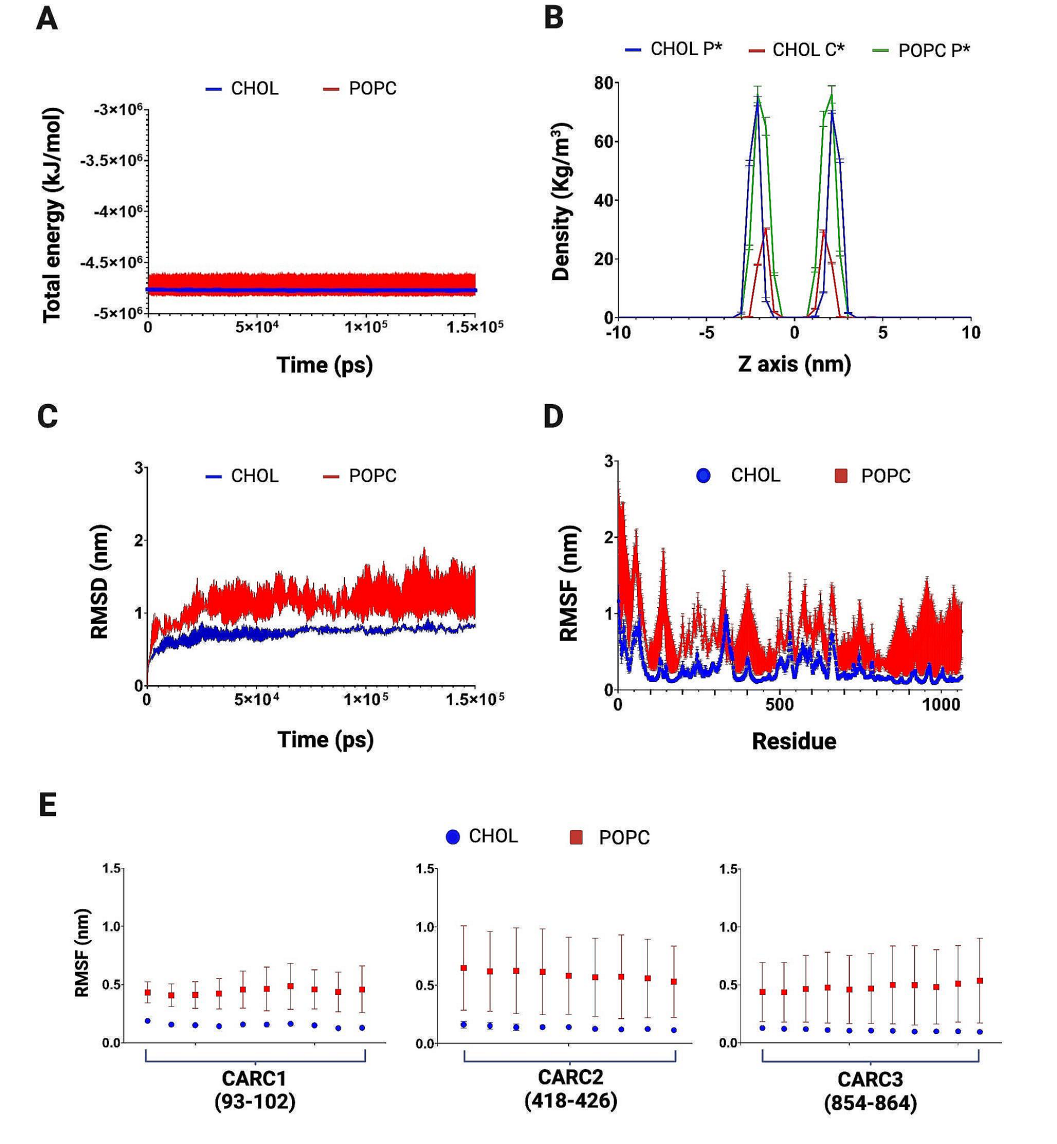
Parameters obtained from molecular dynamics simulations of PMCA1. (**A**) Total energy calculated along 150 ns of simulation for POPC (-4.71×10^6^ ± 919.9 kJ/mol) and cholesterol-containing (CHOL) systems (-4.77×10^6^ ± 36.5 kJ/mol). (**B**) Partial density distribution of the head of lipids along the Z-axis of the simulation box. CHOL P*: Phospholipids of the system with cholesterol; CHOL C*: Cholesterol of the system with cholesterol; POPC P*: Phospholipids of the system with POPC. (**C**) RMSD as a function of time; POPC (1.37 ± 0.047 nm); CHOL (0.71 ± 0.04 nm). (**D**) RMSF as a function of residue number of PMCA1 in the POPC system or in a system with cholesterol; note that the shown model of PMCA1 includes only 1038 residues. (**E**) RMSF corresponding to each of the three CARC motifs found in PMCA1 (mean values ± SD). RMSD: root mean square deviation. RMSF: root mean square fluctuation

From MD simulations data, the total energy during simulations was determined, being of -4.71×10^6^ ± 919.9 kJ/mol and − 4.77×10^6^ ± 36.5 kJ/mol in POPC and cholesterol-containing systems, respectively (Fig. 5A). In addition, to obtain an estimation of the thickness of the membrane under different simulated conditions, we analyzed partial densities of lipids as a function of the Z-axis during the simulations (Fig. 5B). The RMSD and RMSF parameters, both based on the position of ∝-carbon atoms in the protein during the simulations were also determined. RMSD values appear more stable along the time in the presence of cholesterol (Fig. 5C). Global RMSF values observed in the whole protein are shown in Fig. 5D; while a detailed analysis of RMSF for all CARC motifs shows these regions have minor fluctuations when compared with other sites of the proteins (Fig. 5E). A similar analysis was performed for all transmembrane domains of both PMCA isoforms where, as observed also in Fig. 5E, there is noticeable greater data dispersion in TM corresponding to PMCA1 (Suppl. Mat. 1) compared to PMCA4 (Suppl. Mat. 12 and Fig. 8E), possibly indicative of higher flexibility in the former isoform, even in the presence of cholesterol.

**Fig. 6:**
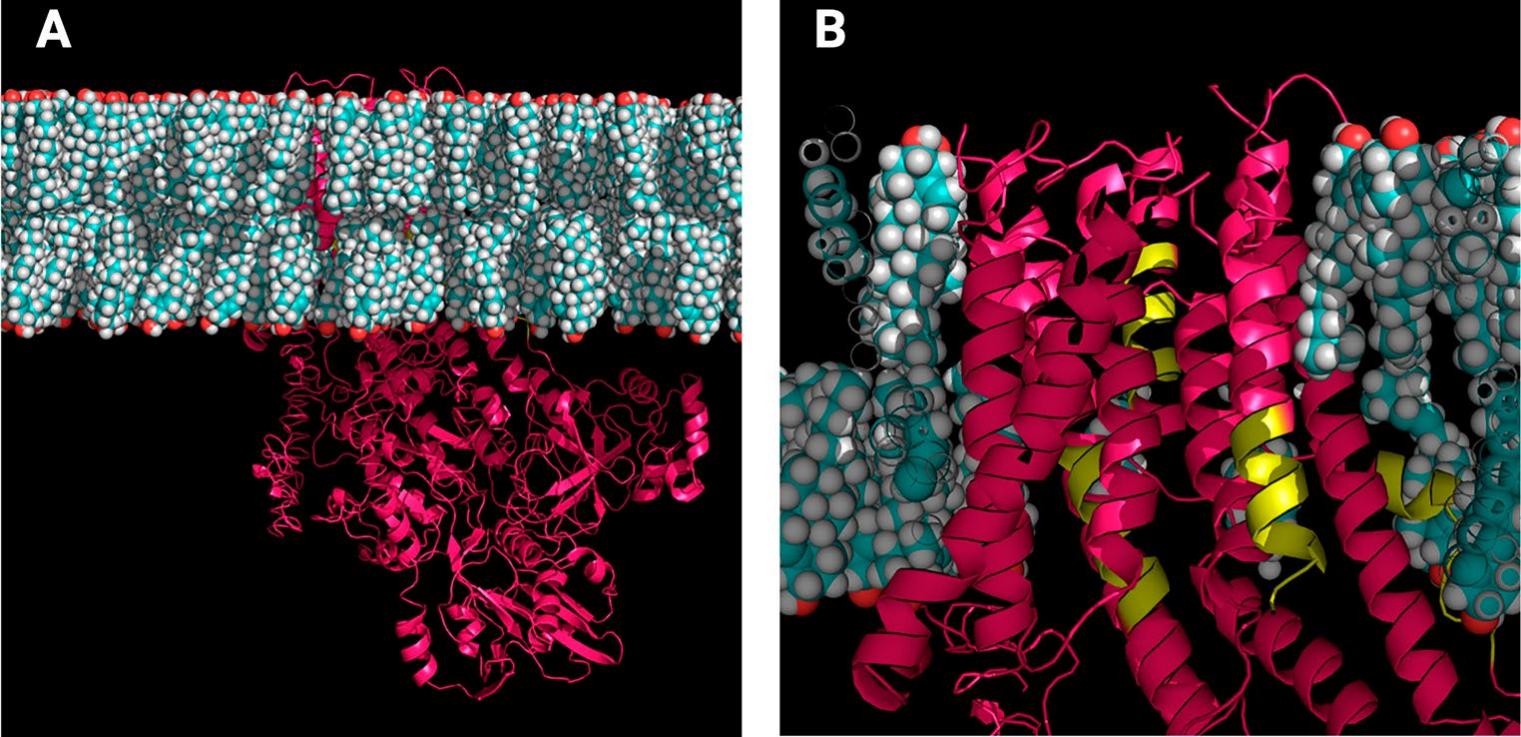
Molecular dynamics simulation of PMCA4. (**A**) Landscape view of simulated systems where PMCA4 (fuchsia) is shown in a membrane containing cholesterol (cyan) and POPC; water molecules, ions, and POPC, were removed from the image. (**B**) Close-up image of the transmembrane domains of PMCA4, where CARC motifs are indicated in yellow

**Fig. 7:**
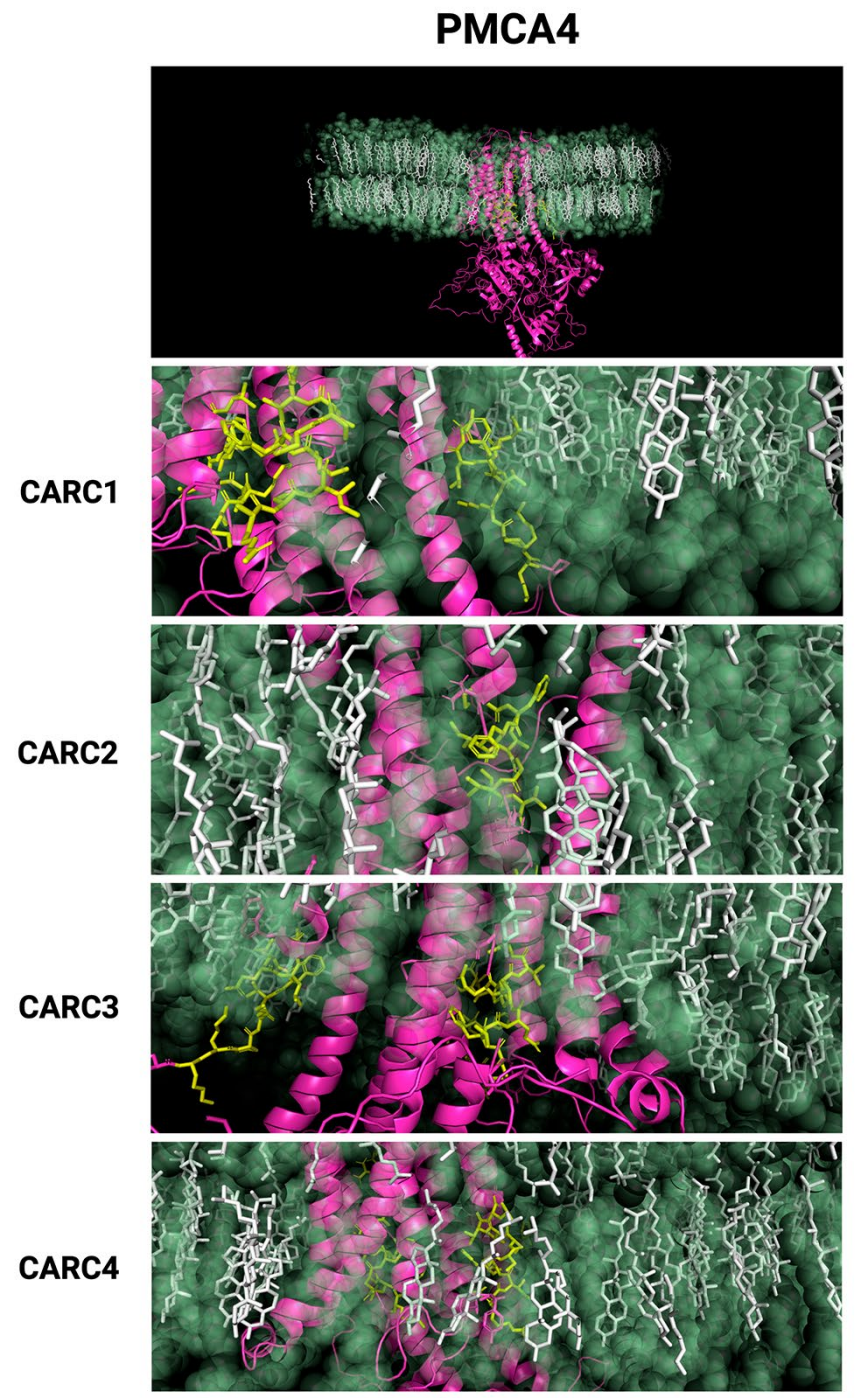
Close-up images of CARC motifs found in PMCA4. The top panel shows the landscape view of CARC motifs. Close-up images of CARC1-CARC4 motifs are shown as green licorice lines, cholesterol molecules are presented as white licorice lines, surrounding POPC molecules are presented as transparent green beads, and non-CARC-containing sequences depicted as fuchsia ribbons

On the other hand, the four CARC motifs predicted for the PMCA4 isoform were observed in the corresponding MD simulation (Fig. 6A). A close-up view shows CARC1 and CARC2 motifs in the interface of the intracellular side of TM1 and TM4, while CARC3 and CARC4 are embedded in TM5 and TM9 (Fig. 6B). Rendered images of CARC motifs of PMCA4 are shown in Fig. 7.

**Fig. 8:**
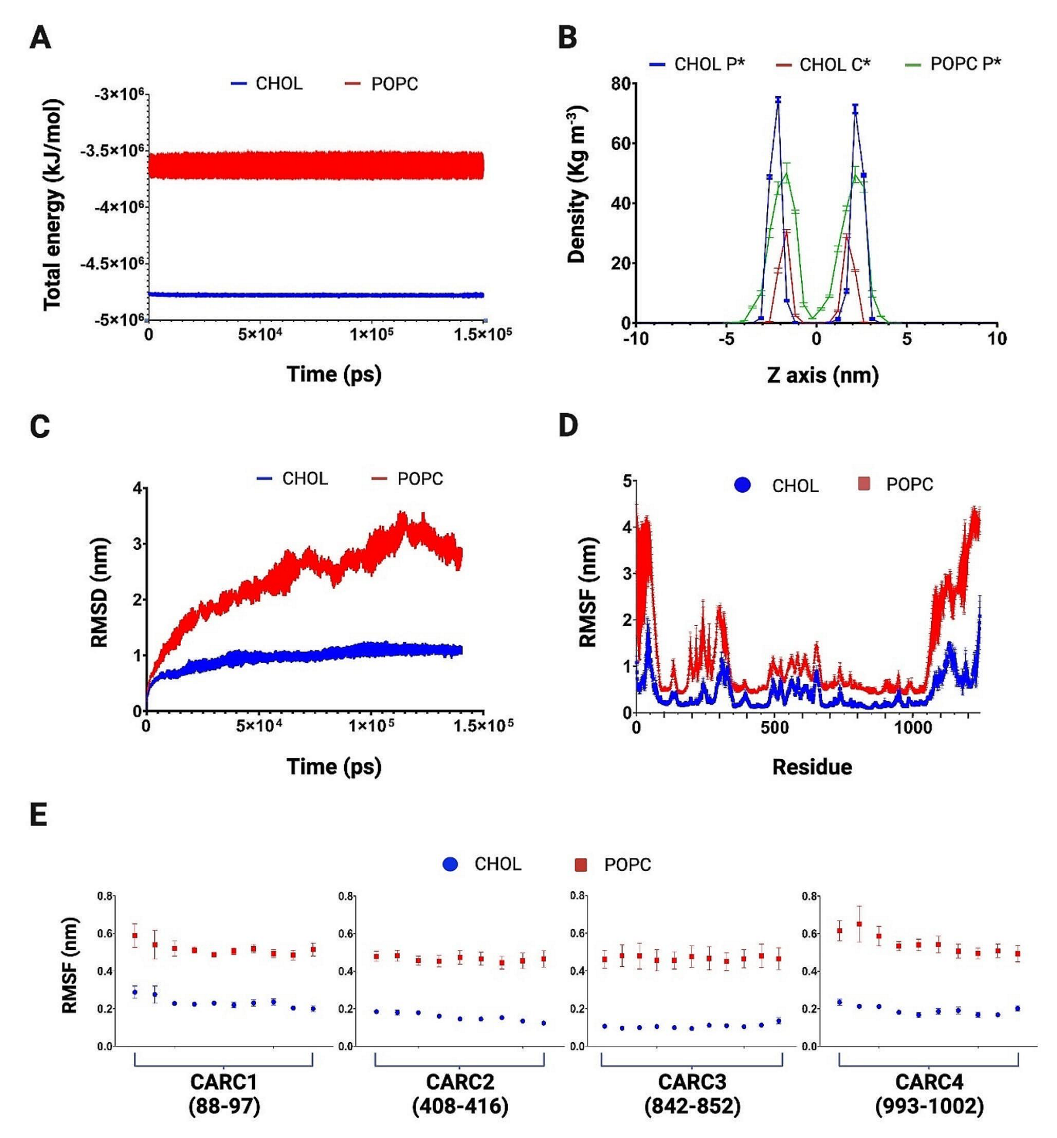
Parameters obtained from molecular dynamics simulations of PMCA4. (**A**) Total energy calculated along 150 ns of simulation for POPC (-3.62×10^6^ ± 1625.8 kJ/mol) and cholesterol-containing (CHOL) systems (-4.77×10^6^ ± 36.5 kJ/mol). (**B**) Partial density distribution of the head of lipids along the Z-axis of the simulation box. CHOL P*: Phospholipids of the system with cholesterol; CHOL C*: Cholesterol of the system with cholesterol; POPC P*: Phospholipids of the system with POPC. (**C**) RMSD as a function of time; POPC (2.39 ± 0.009 nm); CHOL (0.96 ± 0.14 nm). (**D**) RMSF as a function of residue number of PMCA4 in the POPC system or in a system with cholesterol. (**E**) RMSF corresponding to the each of the four CARC motifs found in PMCA4 (mean values ± SD). RMSD: root mean square deviation; RMSF: root mean square fluctuation

The MD simulation performed with this last isoform showed an outstanding difference between the total energy determined when associated with cholesterol or phospholipid, in comparison to values observed for PMCA1 (Fig. 8A). Partial densities of lipids obtained from PMCA4 MD simulation, show similar values to those observed with PMCA1, with slight variations when phospholipids are present (Fig. 8B). RMSD values of PMCA4 appear more stable over time in the presence of cholesterol and even in the presence of POPC, in comparison to those observed in PMCA1 data (Fig. 8C). Interestingly, the RMSF values corresponding to CARCs in both PMCA isoforms tend to be lower and with fewer fluctuations in the presence of cholesterol compared to the sole presence of POPC (Figs. 5E and 8E).

The statistical analysis of RMSF corresponding to TM domains for both PMCA1 and PMCA4 shows significant differences across all TM domains when comparing cholesterol-containing systems to those containing only POPC (Suppl. Mat. 3 and 4).

Additionally, using tools of the VMD program, we generated rendered time-lapse videos enabling the observation of cholesterol molecules in close proximity to the CARC motifs observed from the outer and inner leaflets of PMCA1 (Suppl. Mat. 5 and 6, respectively) and PMCA4 (Suppl. Mat. 7 and 8, respectively).

## Discussion

It is known that a wide range of mechanisms modulates PMCA activity, according to its active role in the homeostasis of calcium in eukaryotic cells contributing to specialized functions in different tissues. In this study, we explored the role that sequences of the CRAC/CARC type, can play in these ATPases. Interestingly, our findings reveal the presence only of CARC sequences in human and rat PMCA isoforms and their regulatory function in the presence of cholesterol. This observation might have noteworthy implications, especially considering that previous estimations of interaction energy of cholesterol and membrane proteins indicate that CARC motifs exhibit a higher affinity for cholesterol than CRAC motifs (-58.0 ± 12.10 kJ mol^− 1^ and − 47.85 ± 10.68 kJ mol^− 1^, respectively) (Fantini et al. 2016). This underscores the potential regulatory role of cholesterol on these ATPases.

The identified CARCs in PMCAs are located in TM domains 1, 4, and 5; while in isoforms featuring a fourth CARC motif (PMCA2 and PMCA4), it is positioned in TM9. CARC1 and CARC2 tend to reside in the inner leaflet of the plasma membrane, while others are fully embedded in TM domains (CARC3, and when present, CARC4). The high similarity in their sequence, nearly 100%, shared by CARC1-CARC3 might suggest an evolutionary relevance for the role they might play in the cell, whereas CARC4 remains enigmatic due to the presence in only two of the PMCA isoforms. In addition, PMCA1 seems to confirm its role as a major housekeeping isoform (Okunade et al. 2004) as indicated by the high homology observed between human and rat species and the well-conserved CARC sequences present in this isoform.

The MD simulation approach is a robust technique offering theoretical support for experimental design and hypothesis testing. Previous research on membrane Ca^2+^-ATPases using MD simulations, has been based on SERCA models, and focused on aspects such as the role of calmodulin in protecting against oxidation (Lushington et al. 2005) and how membrane thickness modulates PMCA activity (Pignataro et al. 2005). More recent works have been based on models of E1, the more stable conformation (with high affinity for Ca^2+^) of PMCA4b, employing short times of simulation (Penniston et al. 2014; Davoudi et al. 2015). Here, based on our previous studies (Santiago-García et al. 2000; Mas-Oliva and Delgado-Coello 2007), we focused on the analysis of the PMCA1 and PMCA4 isoforms and their interaction with cholesterol possibly through CARC domains. The interaction between the different protein motifs is likely dependent on the availability of cholesterol in the cell membrane. In erythrocytes, around 30% of total lipids correspond to cholesterol evenly distributed across the membrane bilayer (Yawata 2003). In contrast, 60% comprises phospholipids that exhibit asymmetric distribution facilitated by different Ca^2+^-activated transporters (Daleke 2008).

The extended simulation times of 150 ns employed in the MD simulations of human PMCA1 and PMCA4, revealed differences in systems containing cholesterol and POPC or POPC alone. The more negative total energy values observed in both PMCA isoforms when cholesterol is present, indicates that the interaction is energetically favorable resulting in a more stable configuration, this effect could also indicate higher affinity for cholesterol regarding the CARCs motifs. In this scenario, the presence of cholesterol might enhance the stability of the PMCA isoform and potentially influence its structure and function. The distinction becomes more pronounced in the case of PMCA4, particularly with the more negative values, suggesting a potential higher affinity for cholesterol likely attributable to the presence of one additional CARC motif. Undoubtedly, understanding the specific motifs involved in the interaction, such as the CARC sequences, as well as variations in their occurrence or positioning, are the focus for future research. Variations in the presence or location of these motifs among different PMCA isoforms may contribute to a more favorable energetic configuration for the protein through cholesterol interactions. Indeed, if a specific isoform features additional CARC motifs, it might exhibit a favorable configuration potentially resulting in a stronger interaction with cholesterol.

The partial density profiles, with the Z-axis zero representing the center of the bilayer, shows symmetric distribution of phospholipids and cholesterol and an estimation of the membrane thickness during the simulations. However, the main difference is observed in the plot depicting the heads of phospholipids in the system with only POPC (POPC P*, cholesterol free system) where the density is lower in PMCA4 compared to PMCA1 (Figs. 5B and 8B). This disparity could be associated with the presence of a fourth CARC motif in the PMCA4 isoform, affecting both the mobility of the lipids in the membrane and the thickness of the surrounding areas.

RMSD values indicated in both PMCA isoforms, a more stable configuration structure in the presence of cholesterol; while MD simulations with only POPC shows a less stable trend over time, thus, indicating that the presence of cholesterol contributes to maintaining the stability of the whole structure along the time (Fig. 5 C). On the other hand, RMSF provides information regarding local conformational changes in the backbone of a protein during the simulation. Besides the expected high fluctuations in the amino and carboxyl-ends of both PMCA isoforms (note that the carboxy-end of PMCA1 is not included in the modeling), we observed that cholesterol provides stability in the whole proteins (Figs. 5D and 8D). This stability tends to be higher in zones where CARC motifs reside, suggesting that they not only provide stability of protein-cholesterol interactions but also might have a major contribution in the formation, perhaps as an anchor point, to support and stabilize lipid raft-type domains and the recruitment of proteins in those structures (Figs. 5E and 8E). In this regard, we performed the analysis of H-bond formation between the PMCA and cholesterol or POPC in all systems studied (data not shown). We found an important increase in the H-bond occupancy being around of 2% on average in the absence of cholesterol, up to 20% for some H-bonds when cholesterol is present. We also observed that in the presence of cholesterol it is possible the formation of H-bonds between positively charged amino acids within CARC motifs and POPC. These results strongly suggest the interaction of CARC motifs with lipids in the membrane, this finding holds relevance as many proteins possessing CRAC/CARC motifs fail to demonstrate such interactions (Fatakia et al. 2020; Taghon et al. 2021). Although MD simulations of Aquaporin 0, have shown through RMSD analysis a good stability regardless the presence of cholesterol, H-bond occupancy was larger and more stable in membranes containing cholesterol (O’Connor and Klauda 2011). While cholesterol-recognition motifs were not initially considered at the time of this work’s reporting (Aquaporine 0 has one CRAC and one CARC motif), their results align somehow with the changes we observed in PMCAs packaging and overall stability, where the presence of cholesterol results in a less flexible and more stable structure.

It is well known that cholesterol provides stability for membrane proteins; for example, different serotonin receptors have up to 10 cholesterol molecules bound in their crystal structures (Saxena and Chattopadhyay 2012; Jafurulla and Chattopadhyay 2017; Sarkar and Chattopadhyay 2021). Moreover, a molecular sensor for cholesterol, represented by a lysine residue (K101 in TM2) of a CRAC motif of the serotonin_1A_ receptor was identified through targeted mutation studies (Kumar et al. 2021). An important fact to keep in mind is that membrane lipids contribute to shape membrane-protein function, but the embedded proteins also influence the dynamics of the surrounding lipids (Grouleff et al. 2015). In this study, the rendered images obtained from MD simulations provided valuable insights into the impact of cholesterol on PMCAs’ stability, as well as the influence of CARC motifs on both protein and membrane structure. In health, the role of PMCA as controlling the fine-tuning of intracellular Ca^2+^ has long been acknowledged, recent evidence suggests that this regulation occurs within specific microdomains through points of contact between the endoplasmic reticulum and the plasma membrane (Krebs 2022). To date, the conditions that trigger a potential imbalance in the calcium homeostasis are still not well understood. The present study supports the view that this fine-tuning might also occur in membrane domains where cholesterol is present.

Previous research from our laboratory has demonstrated the association, particularly of PMCA1 as the most prevalent isoform, with lipid raft domains in the liver tissue (Delgado-Coello et al. 2017), and relevance might be extended to pathological conditions affecting this organ. Moreover, our current findings may shed light on the altered Ca^2+^ homeostasis observed in various diseases. For instance, high PMCA1 transcript levels have been observed in epileptiform hippocampal cells and murine hepatocarcinoma compared to control cells (Bravo-Martínez et al. 2011; Briones-Orta et al. 2021). Moreover, we have shown that cholesterol exerts varying effects on PMCA activity in membranes isolated from different tissues. In membranes from cardiac sarcolemma or erythrocytes partially depleted of cholesterol, PMCA activity is increased. In contrast, in membranes isolated from hepatocytes pre-incubated with 2 mM methyl-ß-cyclodextrin, PMCA activity decreases considerably (Delgado-Coello et al. 2017). Together, these results confirm that the effect of cholesterol on membrane proteins, and specifically PMCA, depends on the cellular context as well as whether PMCA forms part of lipid-raft domains. Regarding PMCA4, that is the main isoform expressed in erythrocytes, it has been shown that acetylated tubulin forms a complex with the ATPase, modulating its activity in close relationship with surrounding lipids therefore erythrocyte deformability is reduced when PMCA activity decreases in hypertensive patients (Monesterolo et al. 2015).

In reference to other pathological conditions where PMCA is involved, it is well-stablished that the absence of PMCA1 in knockout mice leads to lethality, highlighting the essential role of PMCA1 in vital physiological processes. Conversely, the deficiency of PMCA4 in male mice provokes infertility (Okunade et al. 2004). Furthermore, various mutations identified in PMCA2/3 isoforms have been associated with diseases affecting the nervous system or different types of cancer (Krebs 2022). The potential of PMCAs as therapeutic target poses a challenge due to their low abundance, lack of specific ligands, the intracellular location of the protein, and that PMCA inhibitors tend to lack specificity (Strehler 2013). Therefore, targeting them therapeutically requires innovative approaches.

Our findings underscore the existence of interactions between CARC motifs and cholesterol attracting attention to a crucial aspect of membrane dynamics. The potential significance of these findings should be explored in the future. Hopefully, further exploration of these interactions will contribute to unveil novel therapeutic avenues aimed at restoring calcium homeostasis in pathological conditions.

## Conclusions

This study highlights the key role of CARC sequences in the intricate regulation of the PMCA, with cholesterol emerging as a central player. By delving into the analysis of changes in total energy concerning the interaction between PMCA isoforms and cholesterol-containing membranes, we unlock valuable insights into the thermodynamics governing these molecular associations. Total energy changes, encapsulating the sum of potential and kinetic energies, offer a comprehensive view of the energetic landscape associated with conformational changes, protein-lipid interactions, and the overall dynamics of the PMCA-cholesterol interplay. While total energy values guide our understanding of the thermodynamics that cholesterol establishes through a stronger and more stable interaction with PMCA than phospholipids, RMSD and RMSF values give us information about the stability of the system, contributing to the understanding of the dynamic behavior of PMCA.

In this context, the transient formation of lipid raft domains during cell signaling serves as a pivotal mechanism for regulating protein function. It is conceivable that CARC motifs contribute to the formation of these domains trough hydrophobic matching or by facilitating the interaction of cholesterol and membrane lipids. Furthermore, investigating into isoform-specific preferences within these total energy changes, elucidates how distinct PMCA isoforms respond to and interact with cholesterol-rich environments. This understanding is crucial in deciphering the intricate connection between energetic preferences and functional outcomes.

### Electronic supplementary material

Below is the link to the electronic supplementary material.

Supplementary Material 1

Supplementary Material 2

Supplementary Material 3

Supplementary Material 4

Supplementary Material 5

Supplementary Material 6

Supplementary Material 7

Supplementary Material 8

## Data Availability

No datasets were generated or analysed during the current study.

## References

[CR1] Abraham M, Alekseenko A, Bergh C, Blau C, Briand E, Doijade M et al (2023) GROMACS 2023.1. Source code. 10.5281/zenodo.7852175

[CR2] Ansah TA, Molla A, Katz S (1984). Ca^2+^-ATPase activity in pancreatic acinar plasma membranes. Regulation by calmodulin and acidic phospholipids. J Biol Chem.

[CR3] Baier CJ, Fantini J, Barrantes FJ (2011). Disclosure of cholesterol recognition motifs in transmembrane domains of the human nicotinic acetylcholine receptor. Sci Rep.

[CR4] Barrantes (2013). How cholesterol interacts with membrane proteins: an exploration of cholesterol-binding sites including CRAC, CARC, and tilted domains. Front Physiol.

[CR5] Bhardwaj V, Purohit R (2020). Computational investigation on effect of mutations in PCNA resulting in structural perturbations and inhibition of mismatch repair pathway. J Biomol Struct Dyn.

[CR6] Bhardwaj VK, Purohit R (2020). A new insight into protein-protein interactions and the effect of conformational alterations in PCNA. Int J Biol Macromol.

[CR7] Bravo-Martínez J, Delgado-Coello B, Garcia DE, Mas-Oliva J (2011). Analysis of plasma membrane Ca^2+^-ATPase genes expression during epileptogenesis employing single hippocampal CA1 neurones. Exp Biol Med.

[CR8] Briones-Orta MA, Delgado-Coello B, Gutiérrez-Vidal R, Sosa-Garrocho M, Macías-Silva M, Mas-Oliva J (2021). Quantitative expression of osteopontin and key cancer markers in the hepatocarcinoma AS-30D model. Front Oncol.

[CR9] Brodin P, Falchetto R, Vorherr T, Carafoli E (1992) Identification of two domains which mediate the binding of activating PL to the plasma membrane Ca^2+^ pump. Eur J Biochem 204: 939–946. 10.1111/j.1432-1033.1992.tb16715.x 10.1111/j.1432-1033.1992.tb16715.x 1311684

[CR10] Cannarozzo C, Merve FS, Girych M, Bojone C, Enkavi G, Róg T (2021). Cholesterol-recognition motifs in the transmembrane domain of the tyrosine kinase receptor family: the case of TRKB. Eur J Neurosci.

[CR11] Caride AJ, Elwess NL, Verma AK, Filoteo AG, Enyedi A, Bajzer Z, Penniston JT (1999). The rate of activation by calmodulin of isoform 4 of the plasma membrane ca (2+) pump is slow and changed by alternative splicing. J Biol Chem.

[CR12] Caride AJ, Filoteo AG, Penheiter AR, Pászty K, Enyedi A, Penniston JT (2001) Delayed activation of the plasma membrane calcium pump by a sudden increase in Ca^2+^: fast pumps reside in fast cells. Cell Calcium 30:49–57. 10.1054/ceca.2001.0212 10.1054/ceca.2001.0212 11396987

[CR13] Conrard L, Tyteca D (2019) Regulation of membrane calcium transport proteins by the surrounding lipid environment. 9:513. 10.3390/biom9100513 10.3390/biom9100513 PMC6843150 31547139

[CR14] Daleke DL (2008) Regulation of phospholipid asymmetry in the erythrocyte membrane. Curr Opinion Hematol 15:191–195. 10.1097/MOH.0b013e3282f97af7 10.1097/MOH.0b013e3282f97af7 18391783

[CR15] Davoudi S, Amjad-Iranagh S, Zaeifi Yamchi M (2015). Molecular dynamic simulation of Ca^2+^-ATPase interacting with lipid bilayer membrane. IET Nanobiotechnol.

[CR17] Delgado-Coello B, Trejo R, Mas-Oliva J (2006) Is there a specific role for the plasma membrane Ca^2+^-ATPase in the hepatocyte? Mol Cell Biochem 285:1–15. 10.1007/s11010-005-9060-z 10.1007/s11010-005-9060-z 16477375

[CR16] Delgado-Coello B, Montalvan-Sorrosa D, Cruz-Rangel A, Sosa-Garrocho M, Hernández-Téllez B, Macías-Silva M (2017). Label-free surface-enhanced Raman spectroscopy of lipid-rafts from hepatocyte plasma membranes. J Raman Spectrosc.

[CR19] Di Scala C, Fantini J (2017). Hybrid in silico/in vitro approaches for the identification of functional cholesterol-binding domains in membrane proteins. Methods Mol Biol.

[CR18] Di Scala C, Baier CJ, Evans LS, Williamson PTF, Fantini J, Barrantes FJ (2017). Relevance of CARC and CRAC cholesterol-recognition motifs in the nicotinic acetylcholine receptor and other membrane-bound receptors. Curr Top Membr.

[CR20] Epand (2006). Cholesterol and the interaction of proteins with membrane domains. Prog Lipid Res.

[CR23] Fantini J, Di Scala C, Evans LS, Williamson PT, Barrantes FJ (2016) A mirror code for protein-cholesterol interactions in the two leaflets of biological membranes. Sci Rep 6:21907. 10.1038/srep21907 10.1038/srep21907 PMC4768152 26915987

[CR24] Fatakia SN, Sarkar P, Chattopadhyay A (2020). Molecular evolution of a collage of cholesterol interaction motifs in transmembrane helix V of the serotonin_1A_ receptor. Chem Phys Lipids.

[CR25] Fujimoto T (1993). Calcium pump of the plasma membrane is localized in caveolae. J Cell Biol.

[CR26] Gál Z, Hegedüs C, Szakács G, Váradi A, Sarkadi B, Özvegy-Laczka C (2015) Mutations of the central tyrosines of putative cholesterol recognition amino acid consensus (CRAC) sequences modify folding, activity, and sterol-sensing of the human ABCG2 multidrug transporter. Biochim Biophys Acta 1848:477–487. 10.1016/j.bbamem.2014.11.006 10.1016/j.bbamem.2014.11.006 25445676

[CR27] Garcia A, Lev B, Hossain KK, Gorman A, Diaz D, Pham THN (2019). Cholesterol depletion inhibits Na^+^,K^+^-ATPase activity in a near-native membrane environment. J Biol Chem.

[CR28] Gong D, Chi X, Ren K, Huang G, Zhou G, Yan N et al (2018) Structure of the human plasma membrane Ca^2+^-ATPase PMCA1 in complex with its obligatory subunit neuroplastin. Nat Commun 9:3623. 10.1038/s41467-018-06075-7 10.1038/s41467-018-06075-7 PMC6127144 30190470

[CR29] Grouleff J, Irudayam SJ, Skeby KK, Schiott B (2015). The influence of cholesterol on membrane protein structure, function, and dynamics studied by molecular dynamics simulations. Biochim Biophys Acta.

[CR30] Hanson MA, Cherezov V, Roth CB, Griffith MT, Jaakola VP, Chien EY et al (2008) A specific cholesterol binding site is established by the 2.8 Å structure of the human ß_2_-adrenergic receptor in an alternate crystal form. Structure 16:897–905. 10.1016/j.str.2008.05.001 10.1016/j.str.2008.05.001 PMC2601552 18547522

[CR31] Huang J, Mackerel AD (2013). CHARMM36 all-atom additive protein force field: validation based on comparison to NMR data. J Comput Chem.

[CR32] Humphrey W, Dalke A, Schulten K (1996). VMD: visual molecular dynamics. J Mol Graph.

[CR33] Jafurulla M, Chattopadhyay A (2017). Structural stringency of cholesterol for membrane protein function utilizing stereoisomers as novel tools: a review. Methods Mol Biol.

[CR34] Jafurulla M, Tiwari S, Chattopadhyay A (2011). Identification of cholesterol recognition amino acid consensus (CRAC) motif in G-protein coupled receptors. Biochem Biophys Res Commun.

[CR36] Jiang L, Fernandes D, Mehta N, Bean JL, Michaelis ML, Zaidi A (2007) Partitioning of the plasma membrane Ca^2+^-ATPase into lipid rafts in primary neurons: effects of cholesterol depletion. J Neurochem 102:378–388. 10.1111/j.1471-4159.2007.04480.x 10.1111/j.1471-4159.2007.04480.x 17596212

[CR35] Jiang L, Bechtel MD, Galeva NA, Williams TD, Michaelis EK, Michaelis ML (2012) Decreases in plasma membrane Ca^2+^-ATPase in brain synaptic membrane rafts from aged rats. J Neurochem 123:689–699. 10.1111/j.1471-4159.2012.07918.x 10.1111/j.1471-4159.2012.07918.x PMC3493797 22889001

[CR37] Jo S, Kim T, Iyer VG, Im W (2008) CHARMM-GUI: A Web-based graphical user interface for CHARMM. J Comput Chem 29:1859–1865. 10.1002/jcc.20945 10.1002/jcc.20945 18351591

[CR38] Jorgensen WL, Chandrasekhar J, Madura JD, Impey RW, Klein ML (1983) Comparison of simple potential functions for simulating liquid water. J Chem Phys 79:926–935. 10.1063/1.445869

[CR39] Kessler F, Bennardini F, Bachs O, Serratosa J, James P, Gazzotti P, Penniston JT, Carafoli E (1990) Partial purification and characterization of the Ca^2+^-pumping ATPase of the liver plasma membrane. J Biol Chem 265:16012–16019. 10.1016/S0021-9258(18)55499-5 2144292

[CR40] Krebs J (2022). Structure, function and regulation of the plasma membrane calcium pump in health and disease. Int J Mol Sci.

[CR41] Kumar GA, Sarkar P, Stepniewski TM, Jafurulla M, Singh SP, Selent J (2021). A molecular sensor for cholesterol in the human serotonin_1A_ receptor. Sci Adv.

[CR42] Li H, Papadopoulos V (1998). Peripheral-type benzodiazepine receptor function in cholesterol transport. Identification of putative cholesterol recognition/interaction amino acid sequence and consensus pattern. Endocrinology.

[CR43] Lopreiato R, Giacomello M, Carafoli E (2014). The plasma membrane calcium pump: new ways to look at and old enzyme. J Biol Chem.

[CR44] Lotersztajn S, Pavoine C, Deterre P, Capeau J, Mallat A, LeNguyen D et al (1992) Role of G protein βγ subunits in the regulation of the plasma membrane Ca^2+^ pump. J Biol Chem 208: 2375–2379. 10.1016/S0021-9258(18)45889-9 1310315

[CR45] Lushington GH, Zaidi A, Michaelis ML (2005) Theoretically predicted structures of plasma membrane Ca(2+)-ATPase and their susceptibilities to oxidation. J Mol Graph Model 24:175–185. 10.1016/j.jmgm.2005.07.003 10.1016/j.jmgm.2005.07.003 16169758

[CR46] Luz-Madrigal A, Asanov A, Camacho-Zarco AR, Sampieri A, Vaca L (2013) Cholesterol recognition amino acid consensus domain in GP64 fusion protein facilitates anchoring of baculovirus to mammalian cells. J Virol 87:11894–11907. 10.1128/JVI.01356-13 10.1128/JVI.01356-13 PMC3807332 23986592

[CR47] Mas-Oliva J, Delgado-Coello B (2007) Protein stability and the evolution of the cell membrane. Comp Biochem Physiol Part C Toxicol & Pharmacol 146:207–213. 10.1016/j.cbpc.2006.09.007 10.1016/j.cbpc.2006.09.007 17142104

[CR48] Mas-Oliva J, Santiago-García J (1990). Cholesterol effect on thermostability of the (Ca^2+^, Mg^2+^)-ATPase from cardiac muscle sarcolemma. Biochem Intl.

[CR49] Méndez-Acevedo KM, Valdes JL, Asanov A, Vaca L (2017) A novel family of mammalian transmembrane proteins involved in cholesterol transport. Sci Rep 7:7450. 10.1038/s41598-017-07077-z 10.1038/s41598-017-07077-z PMC5547113 28785058

[CR50] Monesterolo NE, Nigra AD, Campetelli AN, Santander VS, Rivelli JF, Arce CA et al (2015) PMCA activity and membrane tubulin affect deformability of erythrocytes from normal and hypertensive human subjects. Biochim Biophys Acta 1848:2813–2820. 10.1016/j.bbamem.2015.08.011 10.1016/j.bbamem.2015.08.011 26307527

[CR51] Mukai M, Glover KJ, Regen SL (2016). Evidence for surface recognition by a cholesterol-recognition peptide. Biophys J.

[CR52] O’Connor JW, Klauda JB (2011). Lipid membranes with a majority of cholesterol: applications to the ocular lens and Aquaporin 0. J Phys Chem B.

[CR53] Okunade GW, Miller ML, Pyne GJ, Sutliff RL, Connor KY, Neumann JC et al (2004) Targeted ablation of plasma membrane Ca^2+^ATPase isoforms 1 and 4 indicates a major housekeeping function for PMCA1 and a critical role in hyperactivated sperm motility and male fertility for PMCA4. J Biol Chem 279:33742–33750. 10.1074/jbc.M404628200 10.1074/jbc.M404628200 15178683

[CR54] Ortega A, Santiago-García J, Mas-Oliva J, Lepock JR (1996) Cholesterol increases the thermal stability of the Ca^2+^/Mg^2+^ ATPase of cardiac microsomes. Biochim Biophys Acta 1283:45–50. 10.1016/0005-2736(96)00072-7 10.1016/0005-2736(96)00072-7 8765093

[CR55] Pacheco J, Domínguez L, Bohórquez-Hernández A, Asanov A, Vaca L (2016) A cholesterol- binding domain in STIM1 modulates STIM1-Orai1 physical and functional interactions. Sci Rep 6:29634. 10.1038/srep29634 10.1038/srep29634 PMC4962086 27459950

[CR56] Pászty K, Kovács T, Lacarabatz-Porret C, Papp B, Enouf J, Filoteo AG et al (1998) Expression of hPMCA4b, the major form of the plasma membrane calcium pump in megakaryoblastoid cells is greatly reduced in mature human platelets. Cell Calcium 24:129–135. 10.1016/S0143-4160(98)90080-X 10.1016/s0143-4160(98)90080-x 9803313

[CR57] Penniston JT, Padányi R, Pászty K, Varga K, Hegedűs L, Enyedi A (2014). Apart from its known function, the plasma membrane Ca^2+^ATPase can regulate Ca^2+^ signaling by controlling phosphatidylinositol 4,5-bisphosphate levels. J Cell Sci.

[CR58] Picazo-Juárez G, Romero-Suárez S, Nieto-Posadas A, Llorente I, Jara-Oseguera A, Briggs M et al (2011) Identification of a binding motif in the S5 helix that confers cholesterol sensitivity to the TRPV1 ion channel. J Biol Chem 286:24966–24976. 10.1074/jbc.M111.237537 10.1074/jbc.M111.237537 PMC3137070 21555515

[CR59] Pignataro MF, Dodes-Traian MM, González-Flecha FL, Sica M, Mangialavori IC, Rossi JP (2005) Modulation of plasma membrane Ca^2+^-ATPase by neutral phospholipids: effect of the micelle-vesicle transition and the bilayer thickness. J Biol Chem 290:6179–6190. 10.1074/jbc.M114.585828 10.1074/jbc.M114.585828 PMC4358257 25605721

[CR60] Rajasekaran R, Priya Doss CG, Sudandiradoss C, Ramanathan K, Purohit R, Sethumadhavan R (2008). Effect of deleterious nsSNP on the HER2 receptor based on stability and binding affinity with herceptin: a computational approach. C R Biol.

[CR61] Rivel T, Ramseyer C, Yesylevskyy S (2019). The asymmetry of plasma membranes and their cholesterol content influence the uptake of cisplatin. Sci Rep.

[CR62] Santiago-García J, Delgado-Coello BA, Mas-Oliva J (2000) Thermal analysis of the plasma membrane Ca^2+^-ATPase. Mol Cell Biochem 209:105–112 10.1023/a:1007182907274 10.1023/a:1007182907274 10942207

[CR63] Sarkar P, Chattopadhyay A (2021). Cholesterol footprint in high-resolution structures of serotonin receptors: where are we now and what does it mean?. Chem Phys Lipids.

[CR64] Saxena R, Chattopadhyay A (2012). Membrane cholesterol stabilizes the human serotonin1A receptor. Biochim Biophys Acta.

[CR65] Sepúlveda MR, Berrocal-Carrillo M, Gasset M, Mata AM (2006) The plasma membrane Ca^2+^-ATPase isoform 4 is localized in lipid rafts of cerebellum synaptic plasma membranes. J Biol Chem 281:447–453. 10.1074/jbc.M506950200 10.1074/jbc.M506950200 16249176

[CR66] Sharpe LJ, Rao G, Jones PM, Glancey E, Aleidi SM, George AM, Brown AJ, Gelissen IC (2015) Cholesterol sensing by ABCG1 transporter: Requirement of a CRAC motif in the final transmembrane domain. Biochim Biophys Acta 1851:956–964. 10.1016/j.bbalip.2015.02.016 10.1016/j.bbalip.2015.02.016 25732853

[CR67] Shull GE, Greeb J (1988) Molecular cloning of two isoforms of the plasma membrane Ca2+-transporting ATPase from rat brain. J Biol Chem 263:8646–8657. 10.1016/S0021-9258(18)68354-1 2837461

[CR68] Sigrist CJA, de Castro E, Cerutti L, Cuche BA, Hulo N, Bridge A et al (2013) New and continuing developments at PROSITE. Nucleic Acids Res 41:D344-D347. 10.1093/nar/gks1067 10.1093/nar/gks1067 PMC3531220 23161676

[CR70] Stauffer T, Hilfiker H, Carafoli E, Strehler EE (1993) Quantitative analysis of alternative splicing options of human plasma membrane calcium pump genes J Biol Chem 268:25993–26003. 10.1016/S0021-9258(19)74484-6 8245032

[CR69] Stauffer T, Guerini D, Carafoli E (1995) Tissue distribution of the four gene products of the plasma membrane Ca^2+^ pump. A study using specific antibodies. J Biol Chem 270:12184–12190. 10.1074/jbc.270.20.12184 10.1074/jbc.270.20.12184 7538133

[CR71] Strehler EE (2013). Plasma membrane calcium ATPases as novel candidates for therapeutic agent development. J Pharm Pharmaceut Sci.

[CR74] Strehler EE, Strehler-Page MA, Vogel G, Carafoli E (1989) mRNAs for plasma membrane calcium pump isoforms differing in their regulatory domain are generated by alternative splicing that involves two internal donor sites in a single exon. Proc Natl Acad Sci 86:6908–6912. 10.1073/pnas.86.18.6908 10.1073/pnas.86.18.6908 PMC297959 2528729

[CR73] Strehler EE, James P, Fischer R, Heim R, Vorherr T, Filoteo A et al (1990) Peptide sequence analysis and molecular cloning reveal two calcium pump isoforms in the human erythrocyte membrane. J Biol Chem 265:2835–2842. 10.1016/S0021-9258(19)39877-1 2137451

[CR72] Strehler EE, Caride AJ, Filoteo AG, Xiong Y, Penniston JT (2007) Plasma membrane Ca^2+^ ATPases as dynamic regulators of cellular calcium handling. Ann N Y Acad Sci 1099:226–236. 10.1196/annals.1387.023 10.1196/annals.1387.023 PMC3873821 17446463

[CR75] Taghon GJ, Rowe JB, Kapolka NJ, Isom DG (2021). Predictable cholesterol binding sites in GPCRs lack consensus motifs. Structure.

[CR76] The Uniprot Consortium (2023). UniProt: the universal protein knowledgebase. Nucleic Acids Res.

[CR77] Toyoshima C, Nakasako M, Nomura H, Ogawa H (2000) Crystal structure of the calcium pump of sarcoplasmic reticulum at 2.6 Å resolution. Nature 405:647–655. 10.1038/35015017 10.1038/35015017 10864315

[CR78] Xiong Y, Antalffy G, Enyedi A, Strehler EE (2009) Apical localization of PMCA2w/b is lipid raft-dependent. Biochem Biophys Res Commun 384:32–36. 10.1016/j.bbrc.2009.04.044 10.1016/j.bbrc.2009.04.044 PMC2731683 19379709

[CR79] Yang J, Zhang Y (2015) I-TASSER server: new development for protein structure and function predictions. Nucleic Acids Res 43:W174-W181. 10.1093/nar/gkv342 10.1093/nar/gkv342 PMC4489253 25883148

[CR80] Yawata Y (2003). Composition of normal red cell membranes. Cell membrane: the Red Blood Cell as a model.

[CR81] Yeagle PL (1991) Modulation of membrane function by cholesterol. Biochimie 73:1303–1310. 10.1016/0300-9084(91)90093-G 10.1016/0300-9084(91)90093-g 1664240

[CR82] Zakany F, Kovacs T, Panyi G, Varga Z (2020). Direct and indirect cholesterol effects on membrane proteins with special focus on potassium channels. Biochim Biophys Acta Mol Cell Biol Lipids.

[CR83] Zámbó B, Várady G, Padányi R, Szabó E, Németh A, Langó T et al (2017) Decreased calcium pump expression in human erythrocytes is connected to a minor haplotype in the *ATP2B4* gene. Cell Calcium 65:73–79. 10.1016/j.ceca.2017.02.001 10.1016/j.ceca.2017.02.001 28216081

[CR84] Zylinska L, Soszynski M (2000). Plasma membrane Ca^2+^-ATPase in excitable and non-excitable cells. Acta Biochim Pol.

