# Supplementary figures and images for: Analysis of cholesterol-recognition motifs of the plasma membrane Ca2+-ATPase
Source: J Bioenerg Biomembr. 2024 Mar 4;56(3):205–19. doi: 10.1007/s10863-024-10010-5 (PMC11116186; doi:10.1007/s10863-024-10010-5)

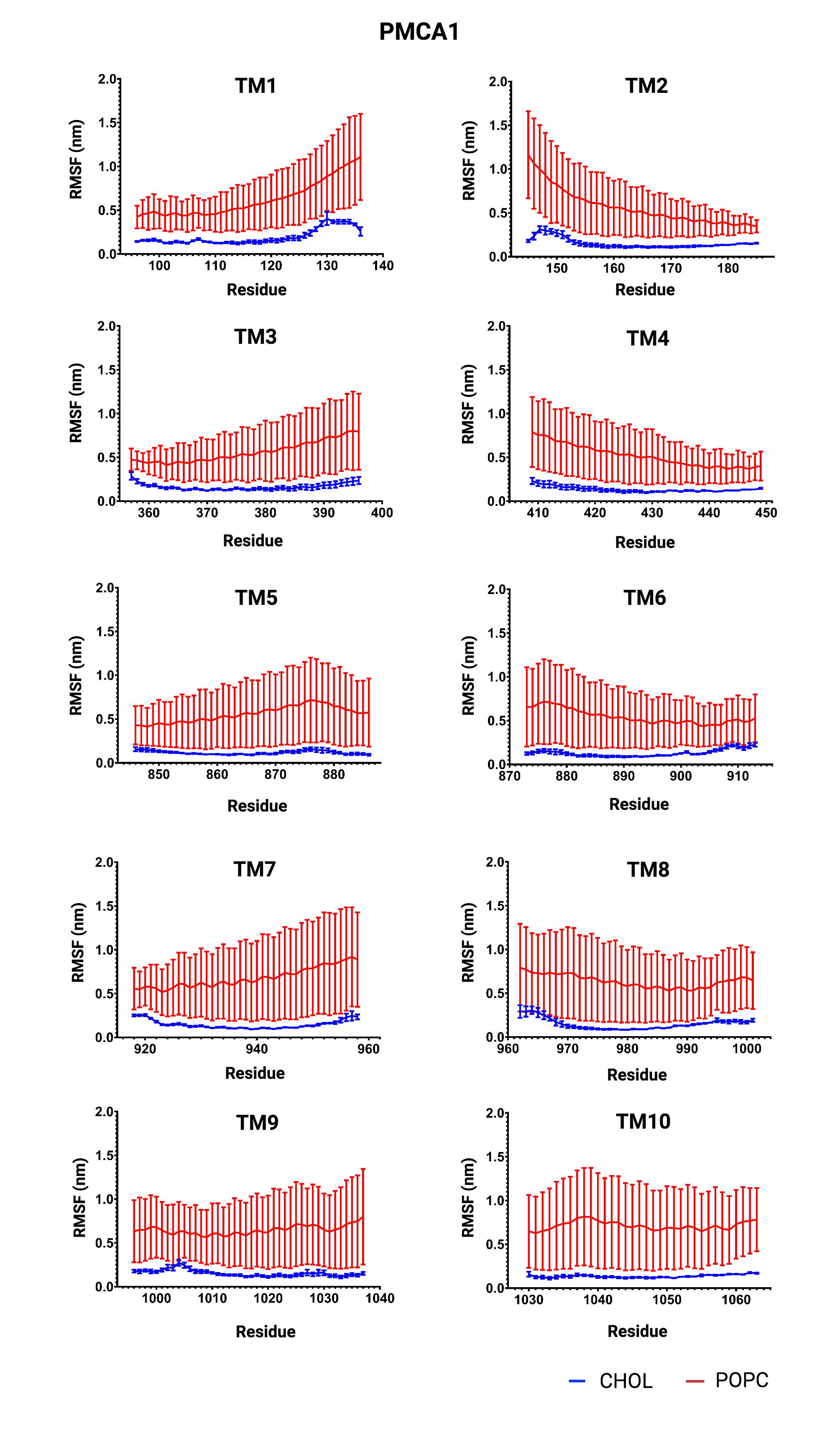

Supplement: Supplementary file 1 — Supplementary Material 1 [file 10863_2024_10010_MOESM1_ESM.jpeg]

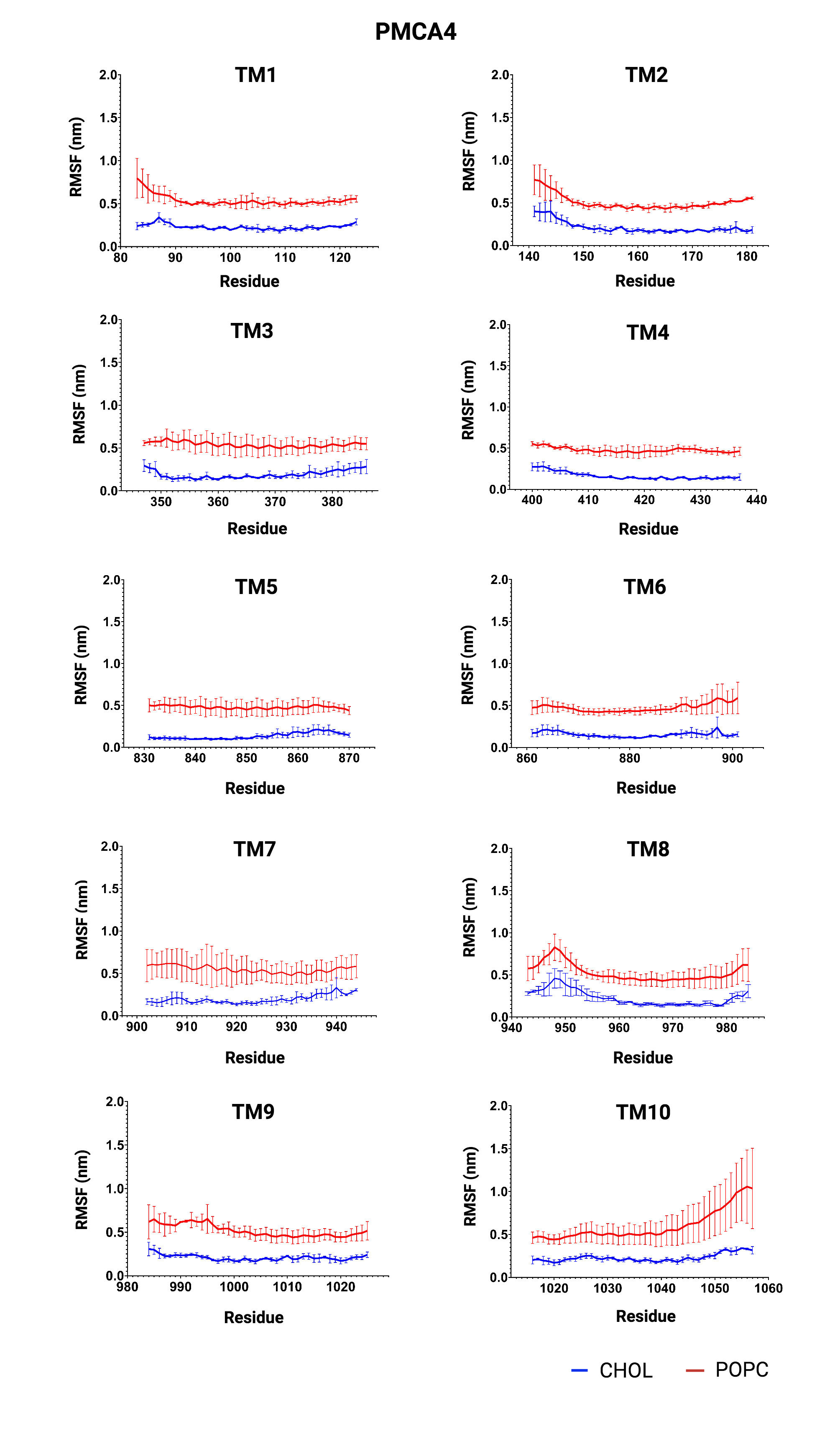

Supplement: Supplementary file 2 — Supplementary Material 2 [file 10863_2024_10010_MOESM2_ESM.jpeg]

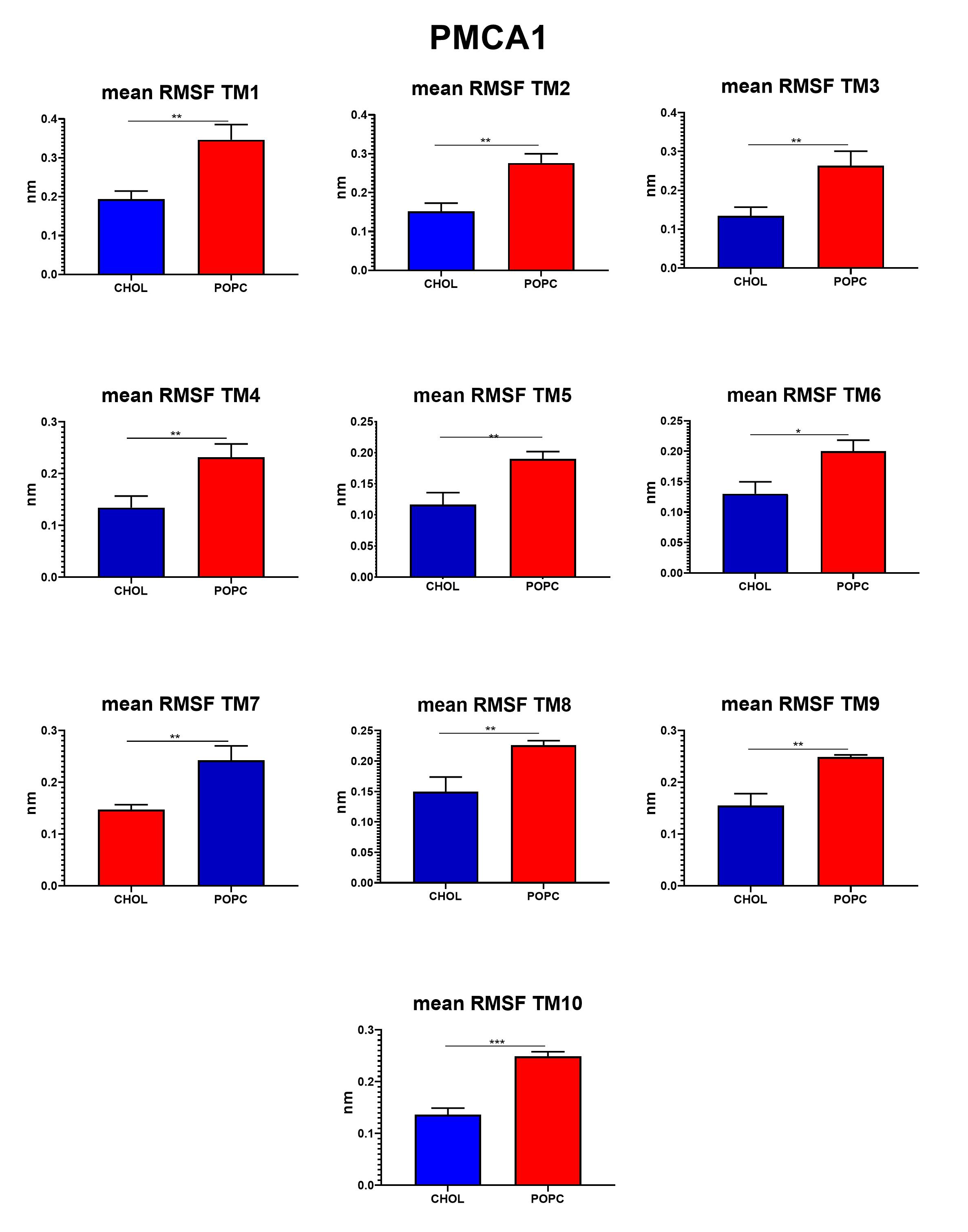

Supplement: Supplementary file 3 — Supplementary Material 3 [file 10863_2024_10010_MOESM3_ESM.jpg]

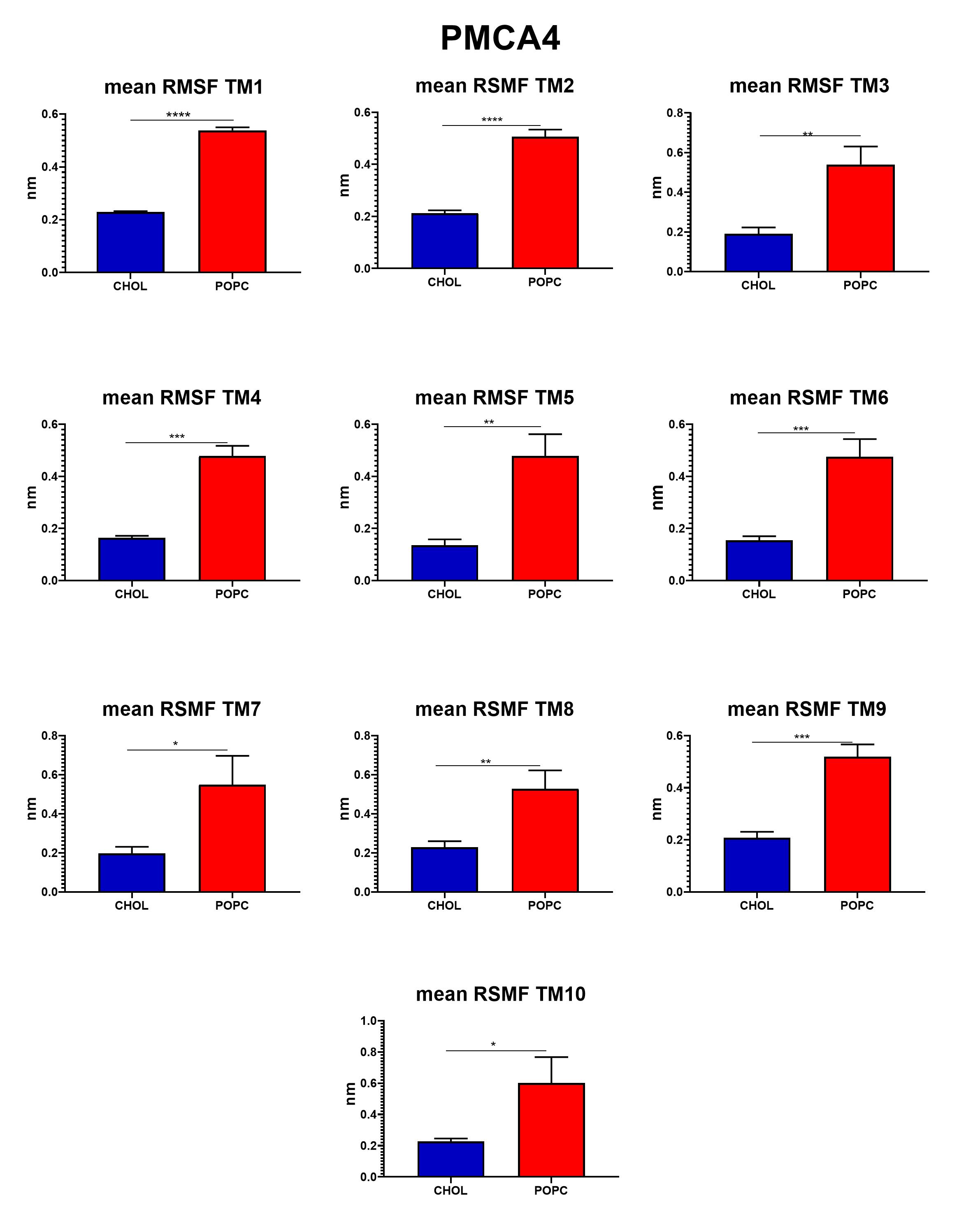

Supplement: Supplementary file 4 — Supplementary Material 4 [file 10863_2024_10010_MOESM4_ESM.jpg]
